# Reassessing therapeutic antibodies for neglected and tropical diseases

**DOI:** 10.1371/journal.pntd.0007860

**Published:** 2020-01-30

**Authors:** Rob Hooft van Huijsduijnen, Somei Kojima, Dee Carter, Hisafumi Okabe, Akihide Sato, Wataru Akahata, Timothy N. C. Wells, Kei Katsuno

**Affiliations:** 1 Medicines for Malaria Venture, Geneva, Switzerland; 2 Chiba-Nishi General Hospital, Chiba, Japan; 3 School of Life and Environmental Sciences and The Marie Bashir Institute, University of Sydney, NSW, Australia; 4 Chugai Pharmaceutical Co, Ltd., Tokyo, Japan; 5 VLP Therapeutics, Gaithersburg, Maryland, United States of America; 6 Global Health Innovative Technology Fund, Tokyo, Japan; 7 London School of Hygiene and Tropical Medicine, London, United Kingdom; 8 Nagasaki University School of Tropical Medicine and Global Health, Nagasaki, Japan; Stanford University, UNITED STATES

## Abstract

In the past two decades there has been a significant expansion in the number of new therapeutic monoclonal antibodies (mAbs) that are approved by regulators. The discovery of these new medicines has been driven primarily by new approaches in inflammatory diseases and oncology, especially in immuno-oncology. Other recent successes have included new antibodies for use in viral diseases, including HIV. The perception of very high costs associated with mAbs has led to the assumption that they play no role in prophylaxis for diseases of poverty. However, improvements in antibody-expression yields and manufacturing processes indicate this is a cost-effective option for providing protection from many types of infection that should be revisited. Recent technology developments also indicate that several months of protection could be achieved with a single dose. Moreover, new methods in B cell sorting now enable the systematic identification of high-quality antibodies from humanized mice, or patients. This Review discusses the potential for passive immunization against schistosomiasis, fungal infections, dengue, and other neglected diseases.

## Introduction

The last three decades have seen a dramatic rise in the use of monoclonal antibodies (mAbs) as therapeutics. By 2017, a total of 78 antibodies had been approved by the US Food and Drug Administration (FDA) or the European Medicines Agency (EMA) [1], with a further 11 approved by the FDA in 2018 [2, 3]. Beyond this, over 570 antibodies are in clinical development [4]. The global mAb market reached US$100 billion annually in 2017 [5], underscoring the considerable economic importance of these medicines.

The success of mAbs starts with the general applicability of the technology used to make them. Antibodies can be developed that have not only high affinity for their targets but also high selectivity, meaning they are less likely to have unwanted side effects and unexpected safety problems. mAbs are particularly good at targeting cell-surface proteins and circulating protein factors; this is in contrast to small molecules, in which cell surface protein–protein interactions have proved difficult to block. In addition, there has been great progress in the development of technology. Early generations of antibodies for human use were developed from mAbs developed in mice, antibodies that were then humanized. Recently, the technology used peptide and antibody display on phages, for which part of the 2018 Nobel Prize in Chemistry was awarded to Sir Gregory P. Winter [6]. More recently, new technologies have been developed to clone antibodies from memory B cells [7] or plasma B cells [8, 9], allowing the isolation of individual antibodies from patients with viral infections—approaches that can be applied to any infectious disease.

A second reason for the popularity of antibodies in recent years is their success rate in clinical development. Once an antibody reaches testing in humans, it has a success rate of 17% to 25% for approval as a new medicine [10], compared with 5% to 10% for small molecules. This success rate is partly due to the exquisite selectivity of mAbs, enabling them to distinguish between closely related molecular targets. In the case of infectious disease, this selectivity can be absolute, since antibodies can be generated that are specific for the invading pathogen and do not cross-react with host tissues. This lack of cross-reactivity with human tissue can be confirmed by immunohistochemistry on both adult and embryonic tissues prior to the start of clinical trials. This is in stark contrast to small molecules, in which sometimes unexpected on- and off-target safety signals are frequently seen in the later stages of clinical development, resulting in expensive late-stage attrition. In addition, antibodies show a relatively narrow range of variation in pharmacokinetic exposure, facilitating early estimation of the human effective dose. This is unlike true xenobiotics, whose metabolism and elimination are usually driven by cytochromes, an enzyme class that is encoded by highly polymorphic genes in a process that is, furthermore, sensitive to drug–drug interactions.

The third major driver for the success of mAbs has been commercial. Many mAbs have been developed in oncology. The most recent success story is the development of the checkpoint inhibitors against targets such as programmed death ligand 1 (PD-L1) and related targets [11]. The dramatic clinical effect in subgroups of otherwise treatment-refractory cancer patients has led to products with prices well in excess of US$10,000 per month [12]. Another recent milestone for an infectious disease was the global approval of ibalizumab, a humanized mAb that targets CD4 (cluster of differentiation 4) for the second-line treatment of HIV-1 infection [13].

Given all this excitement about mAbs, it is pertinent to ask what role they could play in the protection against infection, particularly in the case of diseases of neglected populations. General concerns arguing against their use include the need for simple delivery, the high price, and, indeed, whether there is a need, given the potential for vaccination. In this Review, we show how recent developments in technology have addressed many of these concerns. By looking at the progress of mAbs against specific diseases of neglected patient populations, the key highlights as well as the key development gaps are illustrated.

### Antibodies can provide a simple option for long-term prophylaxis

Most antibodies developed to date are used as therapeutics, targeting cellular mediators that are overexpressed in disease, such as cytokines in rheumatoid arthritis or cell-surface markers in oncology. For the anti-tumor necrosis factor (anti-TNF) antibodies used in rheumatoid arthritis, although there is an initial loading dose to reduce an existing inflammatory signal, the long-term use is to provide prophylaxis against relapse. The first candidate to be approved was infliximab, initially approved for intravenous use at a dose of 3 mg/kg. More recent antibodies have been developed as subcutaneous injections, and adalimumab has taken over as the market leader, with an injection every 2 weeks. Clearly, compliance is linked to the relative ease of delivery of the subcutaneous approach, with a reasonable dose.

In infectious disease, the biggest success story has been the use of antibodies to provide prophylaxis against infection by respiratory syncytial virus (RSV). RSV is responsible for over 30 million episodes of new lower respiratory tract infections, particularly targeting children 5 years and younger, resulting in an estimated 48,000 to 74,500 deaths globally (2015 estimates) [14]. Palivizumab, dosed at 15 mg/kg, is given as a monthly intramuscular injection throughout the RSV season, which is suitable for small babies but would not be appealing for adults. A follow-on antibody—MEDI8897, now in Phase II clinical studies [15]—is 100 times more potent than palivizumab in vitro. It is being developed as a single 50 mg injection to cover the typical 5-month season.

Taken together, these cases illustrate, that after initial proof of concept, it is possible to find second-generation antibodies with more simple routes of delivery and sufficient potency to support relatively infrequent dosing in the field.

### mAbs are becoming sufficiently affordable to be relevant in neglected diseases

A major objection to the use of mAbs in infectious diseases as a therapeutic class is their high price, which is presumed to be a consequence of a high cost of goods. Manufacturing costs are a particularly important attribute for any medication that is developed for diseases of poverty in low- and middle-income countries (LMICs). However, production efficiency of mAbs has increased dramatically over recent decades, and cell-culture expression levels around 4 g/l or even higher are common. A published analysis from a decade ago already noted that the costs of mAb production were dropping from US$300/g to US$20/g when being produced at the 10-tonne-per-year scale, potentially using large, 100,000 L reactors [16]. A more recent analysis has similar estimates [17], which—depending on process and volume—range from US$20/g to US$80/g [18].

For a public health application against an infectious disease in an LMIC, an intramuscular or subcutaneous delivery would be preferable (rather than the intravenous route). The intramuscular route effectively limits the dose; an injection volume of 1.0 ml is probably close to the maximum acceptable in small children, and antibodies are typically only soluble to around 100 mg/ml, which gives a limitation on the total dose of around 100 mg, corresponding to a range of 5 to 20 mg/kg [19]. Taken together, the cost of goods of an injection based on current numbers would be in the range of US$1 to US$8. Benchmarking such costs is difficult, but as a comparison, the recently launched malaria vaccine provides less than 50% protection for 3 injections at around US$5 each. The programmatic cost of protecting a child from malaria for a year in the Sahel using existing low-cost oral medicines has been estimated at US$3.40 by Unitaid [20]. An injectable therapeutic that could protect a child for a season for this price provides a cost-effective option.

The other important factor to manage is the reduction of the frequency of administration, to make the prophylaxis useful in resource-poor settings. mAbs used in therapy are generally IgGs (immunoglobulin Gs), which have plasma half-lives of 20 to 25 days in humans [21, 22]. Such antibodies can be used to provide several months of protection, as in the case of the once-per-season anti-RSV antibody MEDI8897 (nirsevimab) discussed earlier or the anti–calcitonin gene-related peptide (anti-CGRP) fremanezumab, recently approved for prevention of migraines with a dose of 675 mg every 3 months. An alternative to simply increasing the dose would be increasing the half-life of the circulating mAb. Several strategies have been proposed here [23]: Mutations can be made in the IgG Fc (fragment crystallizable) region, which interacts with the receptors responsible for the uptake of antibodies. Such mutations, as for MEDI8897, can significantly extend an mAb’s plasma half-life. Mutations derived from the neonatal Fc receptor (FcRn) increase the strength of the interaction between the Fc region and the Fc receptors under the acidic pH conditions in lysosomes, and as a result, the receptor–Fc complex is recycled back to the cell surface, escaping degradation [24, 25]. This approach was followed for bevacizumab and cetuximab, by mutating Met_428_ to Leu and Asn_434_ to Ser. Discussion about even longer periods of protection has been stimulated by recent results from virally mediated expression of antibodies targeting HIV [26]. These technologies hold out the promise of sustained serum titers of antibodies for more than a year from a single injection, although the technological development is more complicated than for the direct injection of antibodies.

One final objection to the goal of administering monoclonals is that vaccination would instead provide lifelong coverage. However, for some diseases, the development of long-lasting potent vaccines still remains elusive. Many pathogens will not raise an adequate immune response, due to immunosuppression. Finally, antibodies give immediate protection, important during rapidly evolving epidemics or pandemics and for individuals being deployed into zones of high transmission. Antibodies would thus form part of the overall armamentarium, along with drugs, bed nets and insecticides, and vaccines.

### Challenges to developing mAbs against infectious diseases

First and foremost for the development of mAbs is the question of suitable antigen targets. Viruses are the simplest pathogens, and their genomes allow limited means to evade host immunity; therefore, passive and active vaccinations have been most successful in this area. Therapeutic antibodies have been developed and marketed for respiratory syncytial, varicella zoster, vaccinia, and hepatitis B viruses (HBVs) [27, 28]. Historically, immune responses against viruses were considered purely cellular and those against other types of pathogens mostly humoral (antibody based) and less effective. However, nowadays the interplay between both systems is better understood, for instance, with therapeutic opportunities against viruses such as rabies that rely on passive immunization. mAbs that target the initial stages of an infection may provide prophylaxis, and the repertoire of potential antigens for such approaches is greatly helped by the increased availability of genomic and transcriptional information. Passive immunization is especially effective in infections, such as those with the rabies virus, which avoids apoptosis of infected neurons while killing protective T cells so that carriers do not mount an immune response themselves [29]. A recent review [30] lists 21 mAbs (and a nanobody, a single-chain type of antibody produced by camelids) that are currently in clinical trials in the United States. In addition, polyclonal antibodies have been approved in the European Union or the US for cytomegalovirus, hepatitis viruses A and C, and postexposure prophylaxis against measles, rabies, rubella, and tetanus [31]. These approaches appear most suitable for virus infections and for protection against the consequences of snake venom but might also be applied to other types of disease.

There are fewer marketed antibodies that target bacteria, and these target toxins, namely anthrax and *Clostridium* cytotoxin [27]. This paucity may be due to a lack of discovery efforts, a consequence of the historic availability of cheap and effective antibiotics. However, with the worrying rise of antibiotic resistance, the tide is turning [32].

Immunization against protist parasites and fungi is generally ineffective. One problem is that organisms such as *Trypanosoma brucei* (which causes sleeping sickness) and *Plasmodium* (malarial parasites) encode dozens or even hundreds of different surface antigens that are sequentially exposed at their most abundant stage, outpacing the host's immune system.

For some pathogens, clinical trials in infected patients are not possible or desirable. In the case of inhalational anthrax, raxibacumab has been approved using the FDA “Animal Rule” [33]. This provides a route forward in disease areas in which trials with infected human patients are not possible or not ethical.

Recently, the case for discovering mAbs for neglected tropical and other infectious diseases was reviewed [34]. This work took a broad, category-wide approach (viruses, bacteria, parasites) and also included venoms. Snake bites, and the challenge of envenoming, represent a serious health problem, resulting in 81,000 to 138,000 deaths yearly [35], and were recently included in the World Health Organization (WHO) list of neglected tropical diseases. The discovery of mAbs in this area logically follows the long-standing therapeutic successes with passive immunization [36], and the costs and feasibility of developing mAbs were recently reviewed [34, 37][38], including specific clinical development and regulatory challenges [39]. In this Review, we selected diseases prioritized by the Global Health Innovative Technology (GHIT) Fund, an international collaboration between the public and private sectors, supporting collaborations between Japanese and non-Japanese entities to advance global health research and development [40, 41]. The present vaccine discovery statuses for these diseases are listed in Table 1.

**Table 1:** Summary of vaccine and/or passive immunization status for the neglected infectious diseases discussed in this Review.

Disease	Vaccine	Highest discovery status
Dengue	CYD-TDV/Dengvaxia: Efficacy 60%–70%, but only given to persons with confirmed prior infection due to safety issues [42].	Several mAbs, also with in vivo activity: SIgN-3C [43], VIS513 [44, 45], D23-1B3B9 [46–48], and Mab11/mutFc [49].
Fungal infections	No	Two mAbs in past human trials: 18B7 [50] and Mycograb [51]; many preclinical candidates.
HBV	Yes: Energix B and Recombivax HB; 80%–100% efficacy.	E6F6 is in clinical trials [52].
HIV	No	One mAb (ibalizumab [13]) has been approved. Eight mAb checkpoint inhibitors are in human trials, with PRO 140 and Cenicriviroc in Phase III.
Malaria	RTS,S is partially active 30%–50% [53] and in confirmatory trials [54].	mAbs with (prophylactic) in vivo activity, including CIS43 [55, 56] and checkpoint inhibitors.
Schistosomiasis	No	mAbs with in vivo activity: SJ18ε.1 [57–59].
Trypanosomiasis	No	An antibody–drug conjugate was active in vivo [60].
TB	The BCG vaccine (partially active).	Etanercept was trialed (reviewed in [61]); mAbs with in vivo activity: 2E9IgA1 [62], reviewed in [34].
VL	No	Diagnostic antibodies only [63].

Abbreviations: BCG, Bacillus Calmette–Guérin; CIS43, circumsporozoite protein 43; HBV, hepatitis B virus; mAb, monoclonal antibody; mutFc, mutated Fragment crystallizable region; TB, tuberculosis; VL, visceral leishmaniasis

## Schistosomiasis

Human schistosomiasis is caused by infection, mainly with one of 4 species of the blood fluke belonging to Schistosomatoidea. It is estimated that there are over 200 million cases annually in Africa, with two-thirds being *Schistosoma haematobium* and the remainder *S*. *mansoni*. In Southeast Asia, other species such as *S*. *japonicum* and *S*. *mekongi* cause smaller numbers of infections [64]. The mortality associated with schistosomal infections is difficult to calculate. Comorbidities linked to anemia are common, and infection with *S*. *haematobium* can lead to female genital schistosomiasis, which results in increased susceptibility to HIV infection [65].

The life cycle of the parasite is complex, involving both a snail and a human host. In the infective stage, cercariae migrate and penetrate the human host skin to become schistosomula, which then move with the venous circulation, initially to the lungs. After further maturation, the parasite migrates via the heart to the portal circulation and the intestines; from there, eggs are excreted with the feces or, passing through the bladder wall, in urine.

Prophylaxis could therefore either be causal—and prevent entry and survival of the cercariae—or suppressive, by killing the juvenile and adult worms. Clearly, as with the malarial parasites, the more stages of the parasite life cycle that can be inhibited, the more effective the protection will be. The molecular targets best addressed by antibodies are still not clear. Some life cycle differences can be characterized: Schistosomula recovered from the lungs are elongated and more resistant to antibody-dependent, cell-mediated cytotoxicity than newly transformed schistosomula. Praziquantel, the standard treatment for schistosomiasis, acts only against adult worms and has no effects on the juveniles, thus repeat treatment is required to eliminate all the worms in a patient. An ideal treatment would therefore kill both adult and juvenile worms at a submicromolar concentration. This is effectively the target candidate profile for a small molecule inhibitor [66].

No target-product profile (TPP) for prophylaxis against schistosomiasis has been published, but it is possible to start to build one, with the recent characterization of praziquantel for such use in murine models and in pediatrics. Clinically, this treatment reduces infection by 70% to 80% using doses of 40 to 60 mg/kg [67]. In a murine model with infection by *S*. *mansoni* cercariae, the maximum dose of 400 mg/kg killed 96% of all adult worms [68]. However, this murine dose produced a peak exposure of 6 ng/ml, some 10-fold higher than the exposure observed in children at the recommended regimen of 40 to 60 mg/kg [69, 70]. It seems pragmatic to project that an antibody that can reduce the worm burden by 80% in the mouse model, at a dose that is clinically achievable in humans, could be acceptable. The maximally practicable injection of an antibody in children is 2 to 5 mg/kg, and given the relatively consistent allometry between humans and mice for mAbs, this corresponds to a dose of 80 to 200 μg in mice [19].

Target identification for schistosomiasis is not straightforward. Unlike malaria, the vaccine field offers no clues as to useful antibody targets. Two vaccine candidates are currently being tested in humans. The first is based on the fatty acid-binding protein (FABP) Sm-14 (Schistosoma mansoni-14), which is being tested in 2 small, open-label pilot studies in Senegal (NCT03041766 and NCT03799510). A larger 300-participant trial in Uganda, NCT03910972, is being planned for a vaccine based on Sm-TSP-2 (Schistosoma mansoni-tetraspanin-2) but is not expected to report before 2023.

Clues about other possible ways forward, in terms of antigen selection, come from a variety of areas.First, *Schistosoma* are helminths (or flatworms), and the initial immune response is a characteristically strong T helper 2 (Th2) cytokine reaction, which is not seen in viral or bacterial infections and is less pronounced in protozoan infections. The cytokines that are released include interleukin (IL)-5, which drives the production of eosinophils to attack the parasite, and IL-4 and IL-13, which drive the production of immunoglobulin E (IgE). Epidemiological studies in endemic areas suggest that an age-dependent immunity may develop against infection, or against reinfection after treatment [71–73]. There is a good correlation between this protection and the development of IgE antibodies, resulting from Th2 responses [73, 74]. A hybridoma that produces a monoclonal IgE antibody to *S*. *japonicum*, SJ18ε.1, was identified [57]. This mAb was protective in an in vitro, antigen-dependent, cellular cytotoxicity assay with rat macrophages or eosinophils and also in vivo during the early phase of infection [57–59]. It recognizes a 97 kDa antigen, Sm-97 (Schistosoma mansoni-97), identified as paramyosin, a muscle protein unique to invertebrates [75, 76]. The epitope of paramyosin recognized by SJ18ε.1 was determined to be the SJ18ε.1_359−362_ sequence Ile–Arg–Arg–Ala [77]. Injection of mice with paramyosin provides protection against *S*. *mansoni* infection [78]. The *S*. *japonicum* equivalent protein, Sj97 (S. japonicum-97), has also been proposed as a vaccine target [79], although no further development has been reported. Taken together, this suggests that an alternative approach to generating therapeutic mAbs would be to produce IgG mAbs targeting paramyosin and potentially other soluble, nonsurface antigens [78].

Second, beyond the cell-surface proteins, schistosomes also express a large number of glycans as part of their glycoprotein and glycolipid repertoire, and an antibody response against those glycans is mounted by the infected host [80]. In 2 cases, the specific antibodies produced could be identified using B cell cloning [81] or after infection of mice genetically modified to express human antibody repertoires [82]. Interestingly, there has been no systematic analysis of the antibodies produced that target the various stages of the parasite life cycle, although early work on *S*. *mekongi* suggests schistosomula and adult worm extracts would induce a better response [83].

In addition to stimulating a Th2 response, schistosome infection can suppress T-cell activation [84–86]; *S*. *mansoni* uses 2 distinct mechanisms to suppress T-cell activation, resulting in the selective up-regulation of PD-L1 on the surface of splenic F4/80^+^ macrophages. The presence of fatigued or anergized T cells opens up the possibility for cotherapy of low doses of mAbs against programmed cell death protein-1 (PD-1) or PD-L1 with antibodies that reverse the anergy.

## Fungal infections

The treatment of invasive fungal infections (IFIs) remains a major challenge worldwide. There are few broad-spectrum antifungal drugs; the most effective have substantial toxicity concerns, and well-tolerated drugs used prophylactically frequently induce resistance. Even with the best current treatment, the risk for mortality due to an IFI can be higher than 40%. For low-income countries, this figure is substantially worse, as many invasive infections are uniformly fatal without treatment, and estimates of global mortality involving fungal infections are as high as 1.5 million annually [87–89]. In addition, fungal infections are the cause of significant comorbidity and mortality in HIV patients. Modeling studies suggest that optimal therapy could save the lives of 1.6 million HIV patients over a 5-year period [90].

The discussion here will focus on mAbs developed for the following significant fungal pathogens: *Cryptococcus*, *Pneumocystis* and *Paracoccidioides*, and *Candida*. In addition to antibodies that directly target and inhibit the fungal pathogen, mAbs can be directed to checkpoints that control the host immune response. This approach may be particularly useful against fungal pathogens for which sustained infection is characterized by a shift from a protective Th1 or Th17 response to a noninflammatory Th2 response. The clinical use of anti-IL17 in rheumatoid arthritis clearly exacerbates fungal infection [91], although it remains to be seen whether the reverse—stimulating this pathway—has a clinically useful effect.

*Cryptococcus neoformans* predominantly causes opportunistic infection in patients with HIV/AIDS and is responsible for a large burden of AIDS-related disease and death in sub-Saharan Africa [92]. Although the rate of infection has decreased in recent years through greatly improved access to antiretroviral therapy (ART), mortality in infected patients has not declined, demonstrating the failure of antifungal development to keep pace with improved antiviral treatment. Cryptococcosis first manifests as a pulmonary disease but spreads hematogenously to the cerebrospinal fluid and brain to cause meningitis and meningoencephalitis, adding the complexity of crossing the blood–brain barrier to drug development.

The ability of mAbs to protect against a lethal cryptococcal infection in mice was first demonstrated over three decades ago [93], with subsequent work demonstrating the efficacy of various mAbs that target the polysaccharide capsule, an essential virulence factor and the primary host–pathogen interface of *Cryptococcus* infection [94–96]. Among these, mAb 18B7 was evaluated in a Phase I clinical trial [50] and was found to produce a modest reduction in circulating cryptococcal antigen. Further development has been hampered, however, by difficulties in securing funding for a disease for which financial returns are likely to be low—a common problem for neglected infectious diseases. Subsequent studies have examined mAbs that target cryptococcal melanin [97], β-glucan [98], or glucosylceramide ([99–101]; see [102] for additional in vivo and in vitro examples). Host CD40 has also been targeted to stimulate the immune response [103]. In addition to highlighting the potential of mAbs as therapeutics, these studies have demonstrated the diversity of inhibitory actions that mAbs can perform on cryptococcal cells, which can include opsonization and increased phagocytosis, inhibition of fungal growth, capsular polysaccharide release and biofilm formation, antibody-mediated target cleavage, and augmentation of the host response [104–107].

*Pneumocystis*, like *Cryptococcus*, is an important opportunistic pathogen in HIV/AIDS and other immunosuppressed patient populations, with estimates of up to 500,000 cases per year [87]. However, unlike *Cryptococcus*, which is acquired from the environment, *Pneumocystis* spp. are commensals, with different species occurring in the lungs of many mammals. The capacity for endogenous infection and for acquisition from asymptomatic carriers makes *Pneumocystis* an attractive target for immunoprophylaxis. To this end, a variety of programs are aimed at developing a *Pneumocystis* vaccine [108, 109], and passive immunization studies have been initiated using mAbs raised to *Pneumocystis* epitopes. Intranasal administration of 4F11, an mAb that recognizes the *Pneumocystis* kexin-like protein KEX1, was able to prevent transmission of *Pneumocystis* pneumonia from infected to susceptible cohoused mice, demonstrating the feasibility of this approach [110]. Emerging evidence of the importance of B cell–mediated immunity to *Pneumocystis* infection strongly supports further research in this area [111].

*Paracoccidioides brasiliensis*, while less globally prevalent than *Cryptococcus* or *Pneumocystis*, is the most important cause of IFIs in Latin America. mAbs have been raised against the major *P*. *brasiliensis* antigens glycoprotein 43 (gp43) and gp70. In an infected mouse model, anti-gp43 activity, mediated by mAb E3-enhanced phagocytosis of *P*. *brasiliensis* cells, increased interferon-γ production and led to a reduction in the fungal burden [112], while anti-gp70 mAbs significantly reduced fungal colony-forming units and almost completely abolished granuloma formation in the lungs [113]. Antibodies raised against mouse CD25^+^ regulatory T (T_reg_) cells, which control immunity and excessive inflammation, depleted CD25^+^ cells, resulting in less severe tissue inflammation, with reduced mortality in susceptible mice [112]. Again, this demonstrates the potential for mAbs to exert control of fungal infection, either directly by incapacitating the fungal cells or indirectly via modulation of the host response.

*Candida albicans* is the most commonly isolated fungal pathogen globally and is associated with significant morbidity and mortality, particularly in patients with HIV or tuberculosis (TB) infection [114]. A recent paper demonstrated the cloning of antibody genes from B cell cultures derived from patients infected with *C*. *albicans* [115]. These antibodies were capable of stimulating opsonophagocytic macrophage activity and provided protection in a murine model of disseminated candidiasis.

In summary, mAbs offer tremendous potential to augment the antifungal arsenal: Animal models have provided promising results, there is a wide range of potential targets, they have the capacity to both inhibit the pathogen and augment the host response, and it may be possible to target diverse fungal pathogens with an appropriate mAb cocktail or by targeting panfungal antigens. Use of mAbs as adjuvants to existing antifungal drugs also shows promise [116]. However, it should be noted that not all mAbs are inhibitory; indeed, some may worsen infection, species and strain specificity can be an issue, and the mechanistic basis of inhibition by different mAbs can vary dramatically [117]. To date, only 2 mAb treatments have advanced to clinical trials: the previously mentioned 18B7 in *Cryptococcus*, and Mycograb, a heat shock protein 90 (HSP90)-specific antibody fragment, which showed promise for treating *Candida* infections but failed to make it to market due to production difficulties and unresolved safety issues. Antifungal immunomodulation is a complex area, and the field is still emerging. These preliminary studies highlight the potential for exciting new advances in mAb research and application, both for understanding fungal immunity and for manipulating it to tackle life-threatening fungal infections.

## Dengue

Dengue fever is a mosquito-borne viral infection found in tropical and subtropical regions around the world. The dengue virus (DENV) is transmitted by female mosquitoes, mainly of the species *Aedes aegypti* and, to a lesser extent, *A*. *albopictus*. There are 4 distinct serotypes—DENV-1 to DENV-4—of the virus, and all serotypes are presently circulating in endemic areas. DENV infects cells of the human immune system and other cell types, leading to symptoms that include high fever, severe headache, severe pain behind the eyes, joint pain, muscle and bone pain, rash, and mild bleeding. In severe cases, plasma leaks out of the circulatory system, which can be fatal. The global incidence of dengue has grown dramatically in recent decades. One recent study estimated that approximately 390 million people are infected, of which 96 million manifest clinically each year [118]. WHO estimates that, globally, 500,000 people with severe dengue require hospitalization each year and that 2.5% of these infections are lethal.

Antibody-dependent enhancement (ADE) is problematic in dengue infection. The presence of subneutralizing levels of flavivirus cross-reactive serum antibodies (acting against one member of the virus family) may result in an increase in infectivity via ADE of another virus member or serotype, which is observed particularly after secondary dengue infection [119, 120].

Despite decades of effort, there is no effective treatment against dengue. Currently, Dengvaxia is approved by the FDA and is the only licensed dengue vaccine in the world. This is also a live attenuated tetravalent dengue vaccine developed by Sanofi Pasteur [121] that has been approved in several countries. However, interim results from long-term safety follow-up studies demonstrated an increased risk for hospitalization of vaccine-sensitized individuals [122], suggesting that ADE-related concerns are relevant. It has been reported that non-neutralizing levels of anti-DENV antibody can enhance viral entry into host cells by forming a DENV-antibody complex [123, 124]. There is concern that an incomplete antibody against DENVs may cause ADE-mediated severe dengue disease. Hence, there is a need for a safe and highly efficacious dengue therapy or vaccine that provides immunity against all 4 serotypes simultaneously.

mAb therapy is an alternative to vaccines and other therapies against dengue. Many mAbs against dengue from mice and humans have been characterized, and the use of mAbs has also been explored as a therapeutic option. Antibody SIgN-3C, identified by the Singapore Immunology Network, neutralized all 4 dengue serotypes and decreased viremia of all serotypes in mice when given 2 days after infection [43]. A humanized mAb Visterra 513 (VIS513), a pan-serotype anti-DENV developed by Visterra [44, 45], which binds E protein domain III (EDIII) and neutralizes all 4 serotypes of DENV, also showed useful antiviral utility. VIS513 (25 mg/kg or 50 mg/kg) was administered at 5 days post infection in nonhuman primates, and no infectious virus could be detected by either plaque assay or virus isolation after treatment, a finding that was, however, not mirrored by reverse transcription PCR (RT-PCR) findings.

A human challenge model is now available for clinical research in dengue [125]. In this model, the efficacy and safety profile of therapeutic antibodies can be evaluated rapidly in small-scale clinical settings, prior to traditional large-scale field studies with naturally infected patients. To develop mAbs for dengue therapy, it is important to consider an approach that prevents or reduces ADE, and several studies that address this link have been undertaken. A neutralizing human mAb, D23-1B3B9, that targets the fusion loop in domain II showed strong neutralizing activity against all 4 DENV serotypes [46]. However, at subneutralizing concentrations, it also elicited ADE activity in vitro [47]. To reduce the ADE, Injampa and coworkers [48] modified the D23-1B3B9 antibody Fc domain at position N297Q. The modified antibody kept the same cross-neutralizing activity to all 4 serotypes as those of wild-type antibody but lacked ADE activity against all 4 serotypes at subneutralizing concentrations. In another neutralizing mAb, SIgN-3C, 2 leucine to alanine mutations were engineered in the Fc part, abrogating binding to Fc gamma receptors [43]. This mutant Fc version (SIgN-3C-LALA) protected mice, while ADE was completely abrogated. Similarly, Mab11/mutated Fragment crystallizable region (mutFc) (an mAb that is unable to bind to cells with Fcγ receptors [FcγR]) and potentiate ADE have been used as a prophylactic therapy [49]. Passive immunization with this mAb (at 25 mg/kg) reduced viral load and disease progression in nonhuman primates.

Here again, therapeutic antibodies can also be used for prophylaxis, affording immediate and reliable protection.

## Opportunities for other neglected diseases

### TB

TB is a good example of the gap between a pathogen’s prevalence and burden. *Mycobacterium tuberculosis* spreads easily among human populations; presently, about one-third of all humans are infected, and new infections occur in 1% of the population each year. Among these billions of carriers, there are, however, “only” 10 million active TB infections, with 1.3 million deaths in 2016. Thus, the vast majority of carriers keep the pathogen in check. TB exerts 2 levels of immune evasion: one in which it is maintained in a latent state and one in which it breaks free and causes active disease [126]. Several studies have tested antibody therapy in TB, with varying success (reviewed in [127]). Even if an mAb treatment would not be curative, shortening the standard treatment of patients infected with multidrug-resistant (MDR) and extensively drug-resistant (XDR) strains would represent a major advance. In addition, “Checkpoint blockade during chronic TB infection requires further consideration” [128].

### Malaria

The role of protective antibodies in malaria was demonstrated over 40 years ago with the finding that the passive transfer of sera from mice with radiation-attenuated sporozoites delayed the development of infection in other mice [129]. The TPPs for malaria are well described [130, 131]. These include a profile for seasonal malaria chemoprevention, a treatment successfully launched in sub-Saharan Africa in the last 5 years, consisting of a full treatment course of 3 days of amodiaquine and 1 dose of sulfadoxine/pyrimethamine. It is given monthly to children during the rainy season. Antibody therapeutics for malaria could (1) prevent the entry (initial infection) of the parasite, (2) block entry of the sporozoite into the liver cells, (3) block entry of the merozoites into the erythrocytes, or (4) block the uptake of the gametocytes into the mosquito (breaking the transmission cycle).

One difficulty in targeting the merozoites in symptomatic malaria is that the extracellular phase of the pathogen is relatively short-lived and is only a small part of the life cycle. Additionally, the number of merozoites invading erythrocytes is very large (up to 10^12^), compared with the number of sporozoites invading the liver stages (dozens). As such, the most interesting place to intervene with an mAb is at the initial infection of the liver. Currently there are 3 mAbs published with potent activity against the circumsporozoite protein, CSP, and these can reduce the parasitaemia in sporozoite-infected FRG (triple mutant Fah/Raγ2/IL2Rγ) mice that carry a human liver implant [132]. These mAbs include mAb317, cloned from B cells obtained from a patient in the recent RTS,S vaccine trial [133], CIS43 (circumsporozoite protein 43 [56]), and a set that included MGU12 [134], cloned from patients vaccinated with irradiated sporozoites. Antibodies that block the invasion process of the red blood cells by merozoites represent another possible approach. In studies of the merozoite protein RH5 (reticulocyte-binding protein homologue 5) as a potential vaccine, some antibodies were described that could block the cycle of erythrocyte infection [135]. The specific merozoite epitopes are now being characterized, and this could form the basis of a second-generation antibody [136, 137].

A general observation with *Plasmodium* infections is that the human host is unable to mount a sterilizing immune response. One theory is that the parasite can control the T-cell response and induce a state of fatigue or anergy similar to that seen in tumor-invading lymphocytes in immuno-oncology. Several studies in mice have demonstrated that blocking PD-L1 or CTLA-4 (cytotoxic T-lymphocyte-associated protein 4) improves clearance in mice infected with *Plasmodium bergheii* [138], and from this and similar experiments, a strong argument can be made that reversal of this fatigue by mild checkpoint inhibitor blockade may be a way of facilitating the host response [11].

### HIV

mAb therapies have also been proposed for HIV. The case for HIV was recently reviewed [139, 140]. Two such mAbs are in Phase III trials: PRO 140 and Cenicriviroc. PRO 140 targets CCR5 (cysteine-cysteine chemokine receptor type 5) and recently entered Phase IIb/III trials for weekly subcutaneous dosing for monotherapy maintenance [141]. Cenicriviroc is a dual CCR2 and CCR5 antagonist investigated for a number of indications, including HIV infection [142]. As noted earlier, ibalizumab was recently approved as a second-line treatment for HIV treatment [13].

### Hepatitis B

For HBV infection, the hypothesis is that high circulating HBsAg levels prevent a proper immune response. A novel mAb, E6F6, is being evaluated for reducing HBsAg levels in patients [52]. In addition, the HBV S protein is being targeted for the discovery of therapeutic mAbs [143].

### Leishmaniasis

In the case of visceral leishmaniasis (VL), IL-10 and glucocorticoid-induced TNF-receptor–related protein have been considered as mAb targets [144], but there has been no systematic analysis of antigens or reported cloning of B cells from infected patients.

## Future directions and conclusions

Although mAbs have made a massive impact in controlling autoimmune disease, inflammation, and cancer, the relative impact in the world of infectious disease has largely been confined to viral diseases. The use of mAbs to protect against RSV infections and the profile of second-generation antibodies shows that it is possible to obtain mAbs that are sufficiently potent to provide long-term protection with a single intramuscular or subcutaneous injection. With the development of new technologies for cloning antibodies from B cells or plasma cells taken from patients infected with bacteria, viruses, fungi, or even protozoal pathogens, it is possible to quickly obtain fully human antibody collections with potential activity against pathogens in vitro and in vivo.

Technologies for expressing antibodies are now at the stage in which it is not uncommon to see extremely high levels of expression in cell culture, and taken together with the progress in reducing costs of production, the cost of goods for an antibody injection is starting to enter the range of US$1 to US$10, reaching the edge of what is affordable for infectious diseases of neglected populations.

Studies of mutations in the Fc region confirm that mAb half-lives can be extended, and the goal of a single injection to cover an entire season for those infections with seasonality is now a possibility. New technologies with viral delivery offer the promise that a single injection could give protection for even longer periods. For many infectious diseases, we are now seeing the buildup of a portfolio of potential antibodies. In cases in which little progress has been made, a systematic attempt is needed to identify the antibodies resulting from successful control of an infection in patients.

Beyond these basic antibodies, the availability of new monoclonals with anti-infective activity in vivo would open up the door to even more creative options: Bispecific antibodies could be used in key immune cells and could effectively support the natural response to infection. Studies in animal models of chronic malaria infection led to the observation that this results in a reduction in the impact of cytotoxic T cells and modulates the T_reg_ capacity, leading to an “exhausted” or ineffective T-cell response [145]. Clinical trials are already underway that use immune checkpoint blockers for chronic HIV and HBV infection [11]. This has important implications for any infectious disease in which a single infection does not drive a sterilizing immune response. For such diseases, which include malaria, one question for the longer term is whether immune checkpoint inhibition, or interfering with interferon-α signaling, could be used [128].

Two decades ago, one of the biggest challenges of working in anti-infective drug discovery was the need to have new medicines with activities against the widest range of pathogens. In more recent times, the tide has changed, and clinical diagnosis now is such that medicines with a high degree of selectivity and specificity are often preferred—as long as they show good clinical activity. This shift to highly specific medicines favors the use of mAbs. Given the overall rise in interest for new treatments in infectious diseases caused by concerns about antimicrobial resistance, there is a real opportunity now to progress the newly emerging families of mAbs to target infectious diseases of neglected populations. Because of outdated preconceptions about this class of therapeutics, few research funds are being allocated to their discovery, resulting in an egg-and-chicken problem (the absence of conspicuous success driving additional efforts). One of the goals of the analysis that we present here is to promote making additional funds available to pursue the initial discovery of mAbs for neglected diseases.
